# Crucial role of T cells in NAFLD-related disease: A review and prospect

**DOI:** 10.3389/fendo.2022.1051076

**Published:** 2022-11-15

**Authors:** Tianyu Mao, Rui Yang, Yi Luo, Kang He

**Affiliations:** ^1^ Department of Liver Surgery, Renji Hospital, School of Medicine, Shanghai Jiao Tong University, Shanghai, China; ^2^ Shanghai Engineering Research Center of Transplantation and Immunology, Shanghai, China; ^3^ Shanghai Institute of Transplantation, Shanghai, China

**Keywords:** nonalcoholic fatty liver disease (NAFLD), nonalcoholic steatohepatitis (NASH), hepatocellular carcinoma (HCC), CD4^+^ T cells, CD8^+^ T cells

## Abstract

Nonalcoholic fatty liver disease (NAFLD) includes a series of hepatic manifestations, starting with liver steatosis and potentially evolving towards nonalcoholic steatohepatitis (NASH), fibrosis, cirrhosis or even hepatocellular carcinoma (HCC). Its incidence is increasing worldwide. Several factors including metabolic dysfunction, oxidative stress, lipotoxicity contribute to the liver inflammation. Several immune cell-mediated inflammatory processes are involved in NAFLD in which T cells play a crucial part in the progression of the disease. In this review, we focus on the role of different subsets of both conventional and unconventional T cells in pathogenesis of NAFLD. Factors regarding inflammation and potential therapeutic approaches targeting immune cells in NASH are also discussed.

## Introduction

As the prevalence of obesity, diabetes, and metabolic syndrome is becoming higher, the incidence of NAFLD is rapidly rising worldwide (1–4), the global prevalence of which is approximately 25% (1), ranging from 13% in Africa (1) to 42% in southeast Asia (3). NAFLD covers a spectrum of diseases involving nonalcoholic fatty liver (NAFL), which is simple steatosis and tends to have a better prognosis, and NASH, which is characterized by hepatocellular injury and fibrosis, subsequently evolving to cirrhosis and hepatocellular carcinoma (HCC) (5–7). The major cause for liver transplantation has already become NASH in the developed countries (8). NASH-related HCC accounts for 10% to 34% of the known etiologies of HCC (9), and tends to be diagnosed at a later stage which is associated with a worse survival rate than HCC related to viral hepatitis (10). However, current therapies for NASH are limited to losing weight and exercising (11), and no drugs are currently approved for treating NASH (12).

The pathogenesis of NAFLD has not been fully elucidated but a “multi-hit” hypothesis is widely accepted. It begins with lipid accumulation in hepatocytes. Subsequent oxidative stress, increased release of pro-inflammatory cytokines and free fatty acid from adipocytes which causes lipotoxicity collectively drive the progression of liver steatosis and inflammation (13, 14). The liver is a critical immune organ, in which T lymphocyte subsets are important immune-regulated cells. Immune system dysfunction contributes to development and progression of NAFLD (15). It is widely believed that innate immunity particularly macrophages play a key part in promoting liver inflammation in NAFLD. Activation of resident KCs and recruitment of monocytes both contribute to NASH through production of ROS, nitric oxide and cytokines such as TNF, IL-1β, IL-6 and TGF-β (16). There is also crosstalk between macrophages and T cells. APCs which include macrophages are indispensable in the activation of T cells to initiate immune responses while macrophages can also be activated and polarized by cytokines secreted by T cells such as IFN-γ, IL-4 and IL-13 (17). As the role of macrophages has already been reviewed (16, 18) and given the increasing importance of T cells in NAFLD, here we review the inflammation related factors and recent evidence supporting the influence of different T cell subsets on the pathogenesis of NASH and NASH-driven HCC. In addition, we discuss possibilities of interfering with T cell mediated responses as a potential approach for treating NASH.

## Inflammation-related factors in NAFLD

Inflammation is tightly associated with the evolution of NAFLD. Several factors including metabolic dysfunction, oxidative stress, lipotoxicity have been verified to induce inflammation in NAFLD. Several evidence has demonstrated that T cells involve in NAFLD through interacting with factors mentioned above.

### Metabolic dysfunction

Insulin resistance (IR) in insulin-sensitive tissues has been considered as a critical determinant of development and progression of NAFLD (19). This view is consistent with the fact that the severity of NAFLD is related with systemic IR (19) and patients with type 2 diabetes mellitus (T2DM) exhibit higher incidence of NAFLD (20). IR may cause fatty liver through persistent activation of hormone-sensitive lipase after a meal thus inducing adipocyte lipolysis which results in increased free fatty acids (FFAs) in serum (21). Stem cell growth factor-beta (SCGF-β) is associated with the severity of IR in a CRP-dependent manner in male patients with obesity (22). Infiltration of specific subset of T cells contribute to IR in white adipose tissue, thus inducing the exacerbation of NASH (23).

Meanwhile, dysfunction of adipose tissue (AT) featured with recruitment of proinflammatory cells (especially macrophages) in visceral AT (VAT) has been verified as a central part in the development of NASH. Mechanically, activated macrophages may secrete cytokines to induce lipolysis, accelerate the delivery of nonesterified fatty acid to the liver and promote the movement of inflammatory molecules toward liver, thus inducing hepatic lesions (24). Chronic inflammation state mediated by polarized macrophages has been associated with IR (25). When treated with high-fat diet, mice develop IR, hepatic lesions and recruitment of macrophages in AT. What’s more, Fabbrini E et al. has revealed that specific subsets of T cells accumulate in AT from metabolically abnormal insulin-resistant obese individuals and lead to IR *in vitro* through production of cytokines.

### Oxidative stress

Oxidative stress is critical in the pathogenesis of NAFLD. In most patients with NAFLD/NASH the levels or activities of biomarkers of oxidative stress show an increasing trend while a decreased concentrations or activities of antioxidant are observed in liver sample (26). KCs and HSCs have been verified to produce ROS dependent of NADPH oxidase (27) (28).Increased ROS generation has been associated with promotion of cell death, inflammation and fibrosis in liver (14). Meanwhile, immune response triggered by oxidative stress has been proven to facilitate hepatic inflammation in experimental NASH dependent of Th-1 activation of CD4^+^ T-lymphocytes (29). Also, oxidative stress results in an increased level of hepatic leptin which contributes to accumulation of CD8^+^CD57^+^ cytotoxic T cells thus playing a vital role in NASH (30). Obesity-related oxidative stress leads to the recruitment of T cells accompanying NASH, fibrosis along with HCC through inactivation of STAT-1 and STAT-3 phosphatase T cell protein tyrosine phosphatase (TCPTP) and activation of STAT-1 and STAT-3 signaling (31). Abnormal accumulation of ROS induces changes in IR and in lipid metabolism which further contribute to aggravation of NAFLD (26).

### Lipotoxicity

Lipotoxicity is one of the major mechanisms resulting in the development of NASH. Accumulation of triglyceride (TG) which used to be considered as a trigger of inflammation and fibrosis in NAFLD. However, this view has been challenged by convincing researches pinpointing that TG functions as a protective factor against lipotoxicity (32, 33). In MCD-diet fed mice, restricting hepatocyte triglyceride biosynthesis can significantly alleviate hepatic steatosis, but fail to inhibit the progression of lobular inflammation and fibrosis in liver (33). What’s more, accumulation of TG is inadequate to induce IR (34).

Saturated fatty acids (SFAs) especially palmitic acid can trigger liver lipotoxicity mainly through impairing IR (35), GSK-3β-dependent hepatocyte lipoapoptosis (36). Lipotoxic intermediates involve in lipoapoptosis induced by SFAs (37). Lysophosphatidylcholine (LPC) and palmitic acid can trigger the release of extracellular vesicles (EVs) from hepatocytes which further promotes the recruitment of macrophages (38, 39). Meanwhile, fatty acids may participate in liver fibrosis in patients with NASH through modifying T-cell profiles (40). SFAs and monounsaturated fatty acids (MUFAs) alter endogenous antigen presentation to hepatic NKT cells and lead to NKT cell depletion, resulting in further upregulation of inflammatory signaling, IR, and hepatic steatosis (41).

Excess free cholesterol can accumulate in hepatocytes, HSCs, KCs and lead to oxidative stress, dysfunction of mitochondria, apoptosis which result in inflammation, fibrosis and a rising incidence of HCC (14, 42).

T cells are lymphocytes which recognize antigen through T cell receptors (TCRs) which are highly variable. Conventional T cells include CD4^+^ T cells and CD8^+^ T cells, with expression of different TCR co-receptors. Liver inflammation are dominated by CD4^+^ T cells in the early stage, with a subsequent CD8^+^ T cell response (29). In addition to the classical T cell subsets, there are innate-like T cells which include the natural killer T (NKT), gamma delta (γδ) T, and mucosal-associated invariant T (MAIT) cells. The unconventional T cells are recognized as innate immune cells but also show features of adaptive immune cells, the role of which in the pathogenesis of NASH should not be ignored either (43).

## Role of CD4^+^T cells

CD4^+^ T cells can protect the liver from infections but also act a pivotal part in hepatocellular injury and autoimmunity (44). Th1, Th2, Th17, Th22 and regulatory T (Treg) cells are functionally different subsets of CD4^+^ T cells which express distinct cytokines (45). Dysregulation of CD4^+^ T cell function is emerging as a critical pathological factor in the development of NAFLD.

### Th1 cells in NASH

Th1 cells are characterized by their ability to produce IFN-γ, which activates STAT4 and STAT1 in effector cells to exhibit pro-inflammatory effects (46).

There is an increase of hepatic Th1 cells in a NASH model (47). Mice with IFN-γ-deficiency show less liver steatosis and relieved fibrosis compared with wild-type (WT) ones (48). The proportion of Th1 cells in both the peripheral blood and hepatic tissues of NAFLD and NASH patients is elevated compared with healthy controls (49, 50), although the number of Th1 cells does not differ between NAFLD and NASH patients (49). Meanwhile, genes encoding cytokines involved in the T cell activation towards a Th1 phenotype are notably increased in NASH compared with NAFLD patients (51). Moreover, the percentage of Th1 cells producing IFN-γ is positively associated with insulin resistance in obese patients, indicated by an increase of the circulating level of leptin, insulin and HOMA-IR values (52). The proportion of Th1 cells in in visceral rather than subcutaneous adipose is also reported to be significantly correlated with plasma CRP, suggesting the involvement of Th1 cells in obesity-driven inflammation (53).

**Figure 1:**
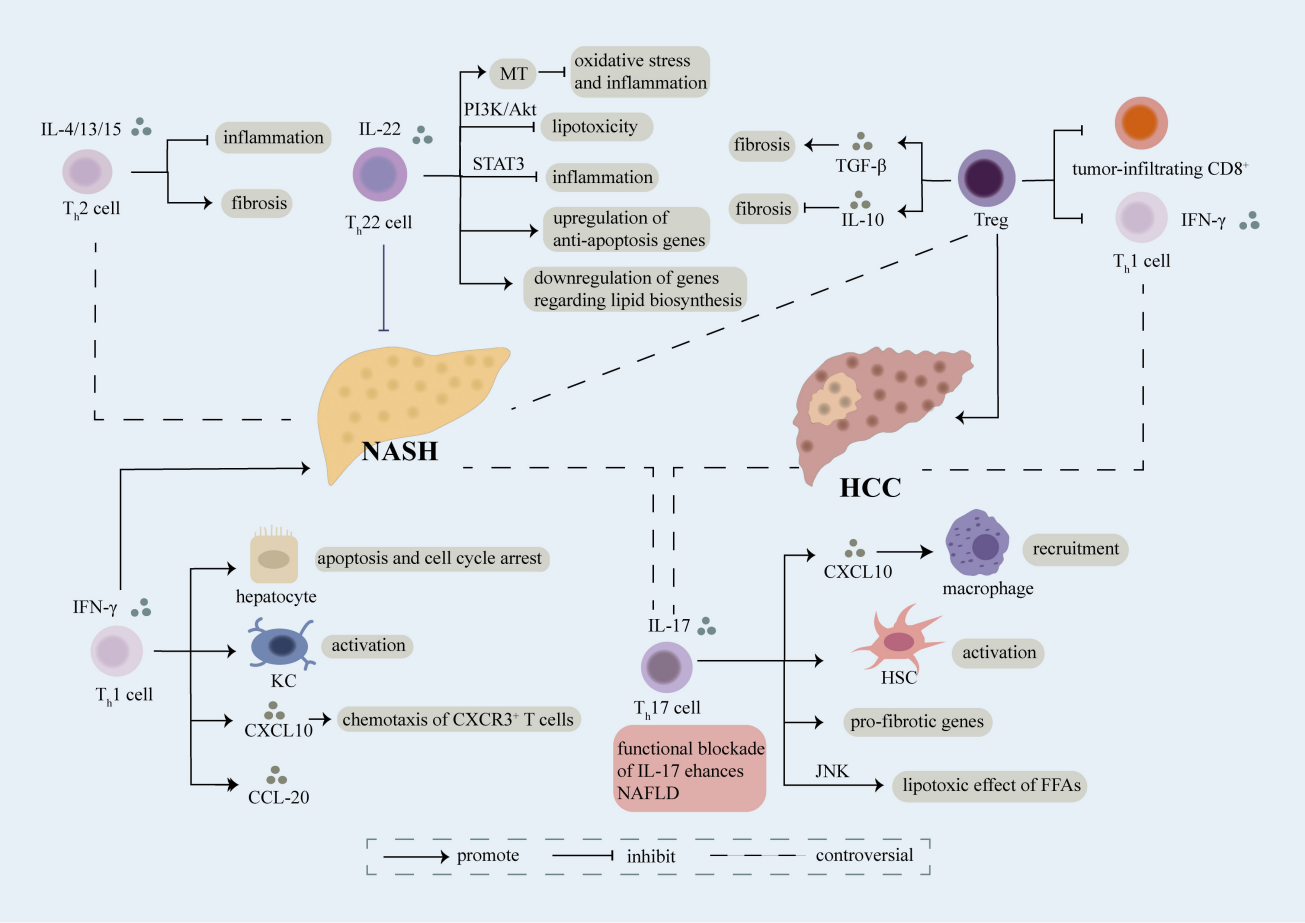
Role of CD4^+^ T cell. Different sets of CD4^+^ T cells including Th1, Th2, Th17, Th22, Treg cells involve in the regulation of NASH and NASH-induced HCC mainly through secretion of cytokines and interaction with other cells. Th1 cells are featured with secretion of IFN-γ, which can further induce apoptosis and cell cycle arrest of hepatocyte, activation of kupffer cells (KCs), production of CXCL20 and chemotaxis of CXCR3^+^ T cells in a CXCL10-dependent manner, thus accelerating the evolution of NASH. The exact role of Th2 cells releasing IL-4/13/15 in NASH remains controversial, on one hand, they play an anti-inflammatory part in NASH, on the other hand, they aggravate fibrosis in NASH. Moreover, there still have some debate over the effect of Th17 cells on NASH. Th17 cells can secret IL-17 which result in recruitment of macrophages *via* releasement of CXCL10, activation of HSC, upregulation of pro-fibrotic genes and lipotoxic effect of FFAs in a JNK-dependent way, thus facilitating the development of NASH. However, several researches have demonstrated that functional blockade of IL-17 enhances NAFLD. Th22 cells characterized by release of IL-22 have been verified as a protective factor of NASH. Th22 cells can suppress lipotoxicity and inflammation through PI3K/Akt and STAT3 pathway respectively. What’s more, MT, an anti-oxidant enzyme, can be upregulated by Th22 cells which further restrains hepatic oxidative stress and inflammatory function of hepatocyte-derived extra cellular vesicles. Meanwhile, Th22 cells involve in upregulation of anti-apoptosis genes and downregulation of genes associated with lipid biosynthesis. Treg cells play a complex role in NASH, they can not only promote fibrosis through release of TGF-β, but also inhibit fibrosis *via* secretion of IL-10. Treg cells lead to exacerbation of NASH-induced HCC through suppression of tumor-infiltrating CD8^+^ T cells and Th1 cells. Th1 cells have been identified as anti-tumor cells through release of IFN-γ, meanwhile Th17 cells have been recognized as pro-tumor cells in NASH-induced HCC. However, a recent study indicated that a predominant Th1 inflammatory pattern contributes to HCC while shifting to a Th17 inflammatory pattern inhibits tumor progression. The exact role of each subset of CD4^+^ T cells in NASH and NASH-induced HCC need further investigation.

IFN-γ can induce hepatocyte apoptosis and cell cycle arrest (54). It can also induce the expression of chemokines such as CCL-20 and their receptors on hepatocytes (55), activate Kupffer cells (56), thus playing a pathogenic role in the liver. Another IFN-γ-induced chemokine CXCL10 can recruit T cells which express CXCR3. Serum CXCL10 levels are elevated in NASH patients while CXCR3 deficiency or deletion of CXCL10 limits liver inflammation, injury and fibrosis (57), and thus the pathogenesis of NASH is reduced in mice (58).Therefore, the effect of IFN-γ deficiency on NASH is partially attributed to the suppressed CXCL10-CXCR3 signaling (**Figure 1**).

### Th2 cells in NASH

Th2 cells are thought to counteract tissue-damaging inflammation, and promote the resolution of inflammation and restoration of tissue homeostasis (59). Th2 cells mainly produce IL-4, IL-5 and IL-13, which activate STAT5 and STAT6 (60). Th2 cells in peripheral blood of NAFLD patients are increased compared with healthy controls (49), while no differences in number of Th2 cells in peripheral blood or the liver have been observed between NASH patients and NAFLD patients or controls (50, 61).

Although Th2 cells may play an anti-inflammatory part in NAFLD (47), it still promotes liver fibrosis in NAFLD, especially under the influence of IL-13. Levels of circulating IL-13 and the expression of IL-13RA2 in the liver are both elevated in NASH patients (62). In a rodent NASH model, liver fibrosis is impeded when IL-13RA2^+^ cells including HSCs are targeted by cytotoxin (62). IL-13 signaling has also been reported to induce lipogenesis and bile-dependent steatosis (63).The production of IL-4, IL-5 and IL-13 can be promoted by IL-33 (64), treatment with which can promote liver fibrosis but also restrict lipid storage in hepatocytes and result in slightly decreased liver injury in a NASH mouse model (65). Therefore, the exact role of Th2 cells in NASH remains unclear and requires further investigation (**Figure 1**).

### Th17 and Th22 cells in NASH

Th17 cells are often recognized as proinflammatory cells, which mainly produce IL-17, IL-22 and IL-23 (43). Th22 cells are characterized as IL-22 producing cells without production of IL-17 (66).

The number of Th17 cells is higher in individuals affected by NASH compared with healthy controls (23, 49, 67) and will decrease 12 months after bariatric surgery (49). Increased infiltration of Th17 cells is observed in the liver of NASH patients compared with NAFLD patients, indicating that Th17 cells may help distinguish NAFLD from NASH (49). While nonsteatotic (CCl_4_-damaged) hepatocytes seem to lack responses to IL-17 signaling, steatotic (metabolically injured) hepatocytes are strongly responsive to IL-17A, which results in upregulation of its receptor IL-17RA, increased secretion of cytokines including IL-6, TNF, CXCL1, and increased synthesis of cholesterol or fatty acids (68). Multiple models have shown that IL-17A can increase hepatic DNA injury, steatosis and fibrosis (23, 69). Mice with deficiency of IL-17A, IL-17F or their receptor IL-17RA exhibit attenuated liver steatosis and injury (69, 70). The application of a monoclonal antibody against IL-17 also significantly reduces lipid accumulation in the liver (71), and attenuates liver fibrosis (23, 47, 71, 72).

Th17-induced hepatic inflammation is mediated by an infiltration of macrophages *via* IL-17-dependent upregulation of CXCL10 (70, 71). The application of an anti-IL-17 monoclonal antibodies remarkably inhibits the activation of Kupffer cells, and reduces pro-inflammatory cytokine levels (71). Likewise, it has been showed that IL-17 exacerbates the lipotoxic effect of FFAs in a JNK-dependent manner (47). IL-17A can also activate HSCs (73) and increase the expression of genes including COL1A1 and ACTA2 (74), consequently exerting a pro-fibrotic effect (**Figure 1**).

IL-17 also plays a critical part in atherosclerosis, which is a more dangerous co-morbidity of NAFLD. It has been reported that IL-17 promotes the development of atherosclerotic plaque *via* increasing the expression of CXCL-1 and adhesion of macrophages to arteries while blocking IL-17 significantly reduces formation of plaque (75), indicating its pro-atherosclerotic effect. IL-17 can also induce secretion of eotaxin by smooth muscle cells, which has a positive correlation with IMT, a marker of atherosclerosis (76).

Conversely, IL-22 may play an inhibitory and protective part in the progression of NAFLD. Administration of recombinant IL-22 significantly attenuates liver injury and steatohepatitis in animal NASH models, possibly through a STAT3-mediated mechanism (77, 78). IL-22 can also attenuate lipotoxicity induced by palmitate to inhibit JNK in a PI3K/AKT-dependent manner (47). However, the effect of IL-22 is not apparent in the presence of IL-17, as phosphatase and tensin homologue (PTEN), the PI3K-AKT antagonist, can be upregulated by IL-17 (47). IL-22 can also upregulate metallothionein (MT), an antioxidant enzyme, to block hepatic oxidative stress and suppress the inflammatory function of hepatocyte-derived extra cellular vesicles, thereby attenuating liver injury and inflammation (79). Besides, IL-22 can upregulate the expression of genes related to anti-apoptosis, including bcl2 and bax, and suppress the expression of scd1, which involves in lipid biosynthesis (80) (**Figure 1**).

Although IL-17 is commonly believed to play a pro-inflammatory, pro-fibrotic and pro-atherosclerotic role in NAFLD-related diseases, several studies have reported an opposite effect, in which functional blockade of IL-17 enhances hepatic steatosis (69, 70) (**Figure 1**) and accelerates the development of atherosclerosis. IL-22 is also widely considered to contribute to atherosclerosis by regulating macrophages and proliferation and migration of VSMCs (81). Moreover, IL-22 therapy increases risk of HCC by activating STAT3, which limits its clinical application (82). In conclusion, the effect of IL-17 and IL-22 is uncertain and remains to be further investigated.

### Treg cells in NASH

CD4^+^ CD25^+^ FOXP3^+^ Treg cells act a pivotal part in establishing immune tolerance and modulating immune homeostasis (83). Treg cells secret IL-10, TGF-β, and IL-35, thus exerting an immunosuppressive effect (84). TGF-β and IL-2 induced STAT5 is essential for their differentiation (85), which can also be regulated by neutrophil extracellular traps (NETs), suggesting that there is interaction between adaptive and innate immunity (86).

Decreased numbers of hepatic Treg cells have been observed in animal models of NAFLD (87–89). Depletion of Treg cells in a NASH mouse model exacerbates disease (90), and aggravates obesity and IR (91, 92), whereas reconstitution of Treg cells can attenuate liver inflammation (88). In addition, NAFLD patients have lower number of circulating and hepatic Treg cells than controls, which decreased more significantly in individuals affected by NASH (49).

The production of oxidative stress, TNF-α and interferon I by KCs and DCs promotes apoptosis of Treg cells (88, 90). This leads to progression of NAFLD to NASH, especially with the exposure to LPS, which can be endogenously produced by the gut microbiota or delivered to the liver (88, 93). Furthermore, adoptive transfer of Treg cells reduces TNF-α signaling induced by HFD and hepatotoxicity caused by LPS (88). Moreover, Treg cells appear to be more susceptible to oxidative stress, consequently altering the ratio of Th17 to Treg cells. A higher ratio of Th17/Treg cells is associated with the severity of liver injury, inflammation, fibrosis, and can help distinguish NASH from NAFLD (88, 94). The function and differentiation of effector T cells and Treg cells can also be regulated by KLF10, which is a TGF-β1-responsive transcription factor (95). In a NASH mouse model, there is a significant decrease of KLF10 expression in effector T cells and Treg cells (96). Accumulation of Treg cells is impaired in mice with KLF10 deficiency, which is associated with IR and NAFLD (96). MIG/CXCL9 can also regulate the differentiation of Treg cells through the JNK pathway and increase the proliferation of Th17 cells, leading to aggravation of NASH (97). Therefore, the imbalance of Th17/Treg cells should be further investigated to provide a non-invasive tool of severity assessment and a potential therapeutic target.

Treg cells are also considered to exert antifibrotic effects, in part owing to their secretion of IL-10 (98) while depletion of Treg cells exacerbates liver fibrosis with marked changes in IL-10 production (99) (**Figure 1**).

Previously adoptive Tregs transfer was considered as a potential therapy for NASH patients (100). But adoptive Tregs transfer exacerbates hepatic steatosis while depleting Tregs may attenuate steatosis in HFD-fed mice (101). Besides, as Treg cells secrete TGF-β, it is widely recognized to be profibrotic in the development of hepatic steatosis and fibrosis (85, 102, 103). These conflicting findings may attribute to the different NASH models used in researches or the opposite function Treg cells have in different stages of NASH (104) (**Figure 1**).

### CD4^+^ T cells in NASH-induced HCC

CD4^+^ T cells are thought to have immune surveillance and antitumor effects, which have been found to recognize tumor cells and initiate their lysis (105, 106). CD4^+^ T cells can also prevent tumorigenesis in models of DEN-induced HCC (107) and mediate elimination of precancerous hepatocytes (108). However, the effect of CD4^+^ T cells on NASH-driven HCC needs further investigation.

Although mesenteric lymph node CD4^+^ T cells have been shown to migrate to the liver and promote hepatic inflammation, a decrease of CD4^+^ T cells in the liver has been reported in NASH models (89), thereby increasing the risk of NASH-to-HCC transition. CD4^+^ T cells are highly susceptible to fatty acid enrichment, especially the linoleic acid, while an accumulation of linoleic acid is observed in the NAFLD liver (89, 109). Linoleic acid impairs mitochondrial function in CD4^+^ T cells, which leads to increased ROS production, caspases activation, and subsequently cell death. Although the numbers are reduced, intrahepatic CD4^+^ T cells are activated and produce IFN-γ, exerting an antitumor effect in NAFLD (89). It is also reported that reduced hepatic CD4^+^ T cells impair the efficacy of the immunotherapy while the administration of N-Acetylcysteine (NAC) can restore CD4^+^ T cells and the antitumor efficacy (110). However, it is advisable to further investigate the effect of linoleic acid on different subsets of CD4^+^ T cells and their role in HCC transition, given their counteracting functions in NAFLD.

Distinct hepatic immunological patterns contribute to the progression or suppression of HCC. A three-dimensional analysis of immune pattern of NAFLD-related HCC shows that a CD8^+^ > CD4^+^, Th1 > Th17 > Th2 pattern is related with tumor progression, while an equilibrium Th1 = Th17 = Th2 pattern in female and a semi-equilibrium Th1 = Th17 > Th2 pattern are associated with remission from HCC (111). It is indicated that a predominant Th1 inflammatory pattern contributes to HCC while shifting to a Th17 inflammatory pattern inhibits tumor progression. However, Th1 cells are widely considered to be anti-tumor and associated with a prolonged overall survival(OS) (112, 113), and an increase of Th17 cells which support cancer development has already been reported, showing the opposite effects (23, 114) (**Figure 1**). Nutrient overload causes DNA damage through recruitment of Th17 cells and increased production of IL-17A by upregulating hepatic unconventional prefoldin RPB5 interactor (URI) (23). Blocking the IL-17A signaling, which can accelerate NASH development, attenuates liver injury and prevents development of HCC (23).

Moreover, Treg cells which increase in peripheral blood and tumor tissues in HCC individuals (115) have been reported to contribute to initiation and progression of cancer in NASH by inhibiting the proliferation and function of Th1 cells which have an effect of cancer immunosurveillance (86). Moreover, Treg cells are thought to be pro-tumorigenic through inhibition of tumor-infiltrating CD8^+^ T-cells (86). Therefore, researchers believe that a decreased ratio of effector CD4^+^ T cells to Treg cells represent a worse prognosis for HCC (116) (**Figure 1**).

Controversial results regarding the effects of CD4^+^ T cells in NASH-driven HCC may attribute to distinct immune cell subsets. It also emphasizes that animal models should be improved to closely mimic human disease (117).

## Role of CD8^+^ T cells

### CD8^+^ T cells in NASH

CD8^+^ T cells can not only be pro-inflammatory cells to accelerate the development of NASH but also function as immune surveillance cells to restrain NASH. Several factors have been verified to influence the abundance, activity or function of CD8^+^ T cells to regulate pathophysiology of NASH. Changes in CD8^+^ T cells have been revealed in both patients suffering from NASH and animal models of NASH. Through transcriptional network analysis, increase of CD8^+^ T cells in blood or liver has been verified in patients with NASH and an experimental model of NASH driven by diet (118). Increased number of CD8^+^ T cells secreting interferon-gamma (IFN-γ), IL-17A and IL-17F are observed in both hepatic microenvironment and peripheral blood in patients suffering from NASH and experimental mice model of NAFLD (50, 61, 70). What’s more, CD8^+^ T cells have been recognized as the dominant intrahepatic immune cells and can activate HSCs in obese model of NASH rather than lean model (119). In patients with NAFLD and HCC, upregulation of receptor for advanced glycation end products (RAGE) on CD8^+^ T cells is revealed which can be a potential biomarker and therapeutic target (120).

**Figure 2:**
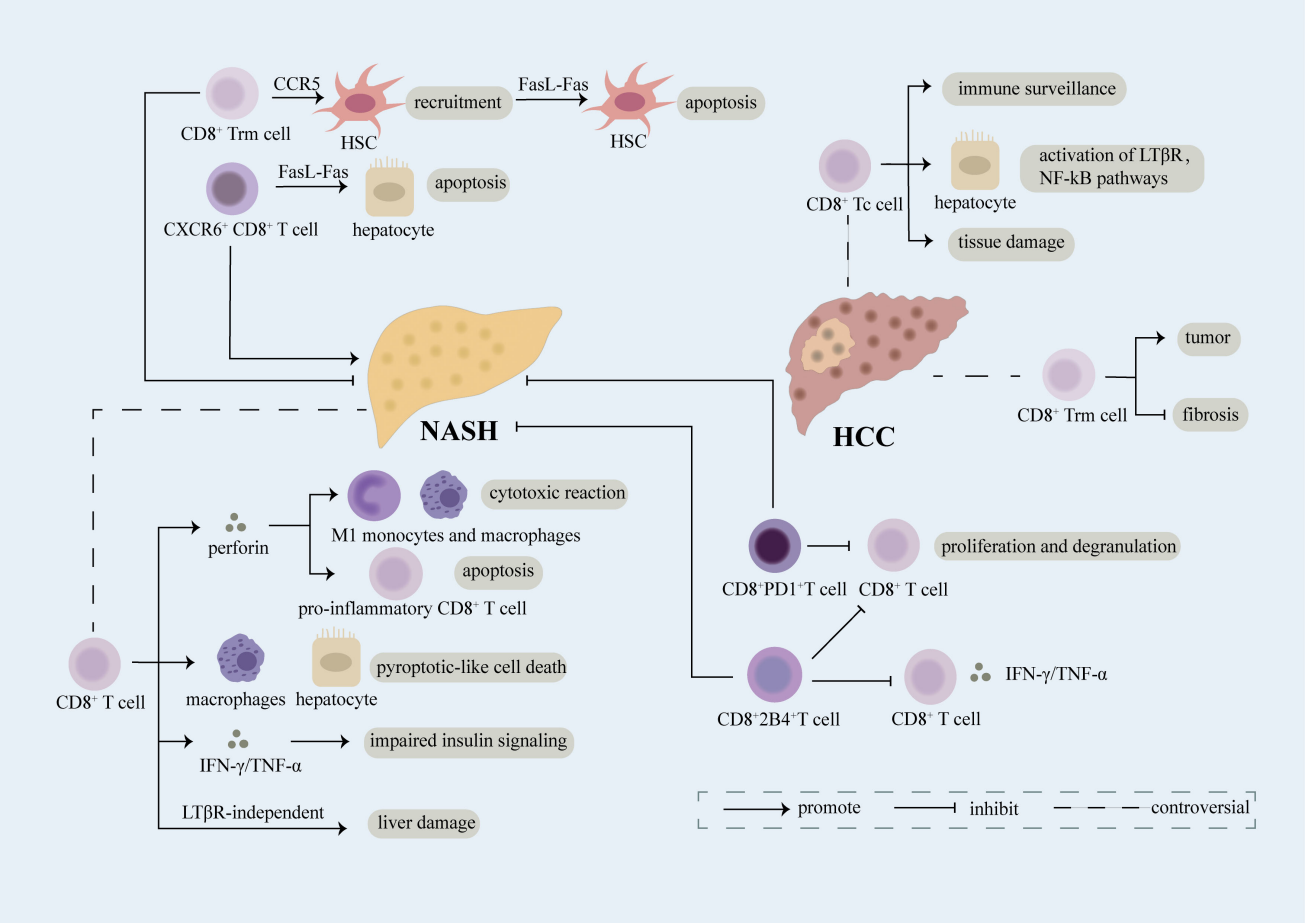
Role of CD8^+^ T cell. CD8^+^ T cells expressing various receptors on the cell surface play a complex role in the evolvement of NASH and NASH-induced HCC. Breaking down of the balance between damage and repairment functions of CD8^+^ T cells may result in the exacerbation of diseases. CD8^+^ Trm cells induce the recruitment of HSC in a CCR5-dependent manner and further lead to apoptosis of HSC through FasL-Fas thus inhibiting fibrosis in NASH. CD8^+^ T cells play an anti-inflammatory part in NASH through secretion of perforin which can not only induce cytotoxic reaction in M1 monocytes and macrophages but also promote apoptosis of CD8^+^ T cells producing pro-inflammatory cytokines. Upregulation of PD1 on CD8^+^ T cells restricts proliferation and degranulation of CD8^+^ T cells, meanwhile, increased 2B4 on CD8^+^ T cells leads to reduced proliferation rate and decreased secretion of inflammatory cytokines, thus relieving liver damage. However, CD8^+^ T cells have been reported as a promoter of NASH. Mechanically, CD8^+^ T cells can induce pyroptotic-like cell death in macrophages and hepatocytes, result in impaired insulin signaling through release of IFN-γ/TNF-α, and lead to liver damage in a LTβR-independent manner. Moreover, CXCR6^+^CD8^+^ T cells accelerate apoptosis of hepatocytes dependent of FasL-Fas which facilitate NASH. CD8^+^ Tc cells, mainly effector cells of CD8^+^ T cells, can not only function as immune surveillance cells to suppress NASH-induce HCC but also act as pro-tumor cells through interacting with NKT cells to activate LTβR, NF-kB pathways and inducing exacerbation of tissue damage. CD8^+^ Trm cells, a kind of CD8^+^ memory cells, have been verified as pro-tumor cells in NASH-induced HCC. However, evidence has shown that CD8^+^ Trm cells can restrict the evolution of fibrosis in NASH which probably limits the transition of NASH into HCC.

CD8^+^ T cells is of great significance in regulating the progression of NASH through various mechanisms. CD8^+^ tissue-resident memory T (Trm) cells, maintained by tissue IL-15, can recruit HSCs in a CCR5-dependent manner and further result in FasL-Fas-mediated apoptosis of activated HSCs which can alleviate and delay the exacerbation of liver fibrosis in mice with NASH (121). When treated with metabolic stimuli (including acetate and extracellular ATP), CXCR6^+^ CD8^+^ T cells function as auto-aggressive cells in a way independent of MHC-class-I, which is induced by increased calcium influx and leads to the upregulation of FasL and apoptosis of hepatocytes thus promoting NASH (122–124). In choline-deficient high-fat diet fed mice, CD8^+^ T cells and NKT cells are found to aggravate the progression of NASH *via* interactions with hepatocytes, during the process of which CD8^+^ T cells participate in liver damage in a LTβR independent manner (125). CD8^+^ T cells can produce perforin, a regulator of liver inflammation, which restrains the development of NASH. Mechanistically, perforin exhibits cytotoxic toward bone marrow-derived M1 monocytes and macrophages and induces apoptosis of CD8^+^ T cells which restrains the production of pro-inflammatory cytokines in MCD-fed mice (126). In an IFN-I dependent pathway, pathogenicity CD8^+^ T cells which result in glucose dysregulation through aggravating hepatic IR and gluconeogenesis are upregulated, thus leading to the development of NAFLD. Cytokines like IFN-γ, TNF-α produced by pathogenicity CD8^+^ T cells probably involve in the process (127). In antigen peptide transporter 1 (TAP1(-/-)) mice which restrict the generation of CD8^+^ T cells, chronic intake of fructose doesn’t result in the development of IR or NAFLD and exhibits a delay in the expression of NAFLD-related genes. This finding supports that CD8^+^ T cells involve in the onset and evolvement of NAFLD (128). What’s more, leptin may trigger macrophages and hepatocytes death in a pyroptotic manner dependent of CD8^+^ T lymphocytes in NAFLD progression (129) (**Figure 2**).

Regulation of abundance, activities or function of CD8^+^ T cells is related to evolvement of NASH. Differential regulation of inhibitory T Cell receptors PD1 and 2B4 on CD8^+^ T cells has been associated with immune tolerance required by liver in NASH. In mice treated with western diet, PD1 is upregulated on liver and peripheral CD8^+^ T cells, 2B4 is augmented specifically in liver rather than periphery on CD8^+^ T cells. Upregulation of PD1 on CD8^+^ T cells restricts proliferation and degranulation of CD8^+^ T cells, meanwhile, increased 2B4 on CD8^+^ T cells leads to reduced proliferation rate and decreased secretion of inflammatory cytokines like IFN-γ and TNF-α, thus relieving liver damage (130) (**Figure 2**). In patients with NASH, suppression of CD8^+^ T cells induced by polymorphonuclear neutrophils (PMNs) which probably leads to impaired immune-surveillance of liver damage, and further facilitates the development of NASH (131). However, dendritic cells restrain the aggravation of NASH partly through limiting the accumulation of CD8^+^ T cells (132). In a NASH mice model, knocking down of mineralocorticoid receptor (MR) downregulates CD25 activation marker on the surface of CD8^+^ T cells which alleviates NASH (133). In MCD-induced NASH, recruitment of CD8^+^ T cells is verified, however, the level of activated CD8^+^ T cell is maintained demonstrating that effector T cells do not play an irreplaceable role in hepatic inflammation in this model (29). When treated with FFA, the expression of PD-L1 which can limit CD8^+^ T cells’ damage toward hepatocytes is upregulate through ROS/ZNF24 pathway, thus rescuing FFA induced injury of hepatocytes (134).

Recently, studies have revealed accumulation and activation of macrophages can be triggered by activated CD8^+^ T cells in adipose inflammation, meanwhile, CD8-independent adipose inflammation is associated with systemic metabolism (135, 136). Considering the tight connection between NASH and adipose inflammation, it probably be a new insight into pathogenesis of NASH.

In a whole, the exact role of CD8^+^ T cells under diverse circumstances in NASH and its interaction with other cells and cytokines need thorough and critical researches to crystallize. The mechanism underlying regulation of CD8^+^ T cells in NASH deserve further investigation.

## CD8^+^ T cells in NASH-induced HCC

### CD8^+^ Cytotoxic T cells in NASH-induced HCC

CD8^+^ cytotoxic T cells (Tc cells) are the main killers of pathogens and cancer cells (137). In an MHC-I dependent manner, they can recognize antigens and further induce cytotoxic process in infected and cancer cells through production of cytokines, secretion of cytotoxic agents (perforins and granzymes), direct contact with cells (43).

The exact role of Tc cells in NASH-related HCC remains controversial. Several studies have demonstrated that Tc cells can accelerate NASH-related HCC. Activated Tc cells are verified to augment in NASH-related HCC in mice model fed by CD-HFD (125). Enrichment of signatures regarding T cells, cytotoxic cells, and macrophages has been found in part of patients with NASH-related HCC (138). The recruitment and activation of Tc cells may partly attribute to STAT-1 or STAT-3 signaling which can promote the expression of T cell chemokines including CXCL9 (31). However, in obesity, STAT-3 signaling can induce HCC in a way independent of recruitment of T cells and evolution of NASH and fibrosis (31). Preventive depletion of CD8^+^ T cell in mice suffering from NASH can significantly inhibit the incidence of HCC, suggesting that with defective immune surveillance functions, hepatic CD8^+^ T cells accelerate HCC in mice with NASH (139). What’s more, CD8^+^ T cells and NK cells probably lead to the activation of LTβR and canonical NF-κB signaling in hepatocytes which facilitates the transition of NASH to HCC. Ablation of CD8^+^ T cells can alleviate liver damage and reduce HCC prevalence (125) (**Figure 2**).

However, Tc cells also function as a critical part of immunity surveillance which restrain the growth of cancer cells (140) (**Figure 2**), inactivation and impairment of Tc cells has been associated with development of HCC. Compared to HCC induced by HBV/HCV, HCC driven by NASH shows a weaker immune response to tumor specific antigen (TAA) owing to the fact that CD8^+^ T cells with strong expression of CTLA-4 is high (141). Tc cells can not only eradicate established tumors but also restrain the early development of cancer (114). Prolonged inflammation and fibrosis in NAFLD can result in the enhancement of immunoglobulin-A-producing (IgA^+^) cells expressing PD-L1 and IL-10 in the liver which can directly inhibit Tc cells in liver, in turn, lead to the development of HCC (114). Antigen-specific CD8^+^ T lymphocytes are impaired by accumulated macrophages in the liver environment thus inducing the development of NAFLD-related HCC (142).

### CD8^+^ memory cells in NASH-induced HCC

Once the infected or malignant cells are cleared, most Tc cells undergo apoptosis, leaving behind memory cells. When secondary exposure to antigens, CD8^+^ memory cells are able to proliferate promptly, gain effector function rapidly and localize to peripheral sites of infection (143).

Recently, several researches have revealed the complex role of CD8^+^ Trm T cells, a unique subset of memory T cells which persist in tissues, in NASH-induced HCC (144). In Ncoa5^+/-^ mouse model of HCC, preneoplastic livers of which are similar to livers of NASH, CD8^+^ Trm cells functioning as pro-tumor cells augment (145). However, as mentioned above, CD8^+^ Trm cells protect against fibrosis in NASH (121), which may further restrain the transition from NASH to HCC (**Figure 2**).

There still have some debates against the role of CD8^+^ T lymphocytes on the transition from NASH to HCC, owing to the fact that CD8^+^ T lymphocytes may participate in multiple stages in the process and animal models used in diverse experiments are not exactly the same. An in-depth investigation is needed which may better guide immune regulatory interventions in patients with HCC.

Also, abnormal CD8^+^ T cells have been associated with insensitivity of patients suffering from NASH-induced HCC to immunotherapy. In a mice model of NASH-related HCC, abundance of CD8^+^PD1^+^ T cells augments, however, anti-PD1 immunotherapy fails to restrain the progression of HCC and even results in a significant increase in the incidence of HCC (139). Mechanically, CD8^+^PD1^+^ T cells featured with an upregulation of effector and exhaustion markers and a decreased proliferative capability are deficient in immune-surveillance functions and instead cause tissue damage which can be partially restrained through PD-1 signaling (139, 146). Meanwhile, it has been found that patients with NASH-driven HCC are less sensitive to anti-PD1 or anti-PDL1 treatment in comparison to patients with other etiologies (139). Impaired response to immunotherapy in NASH-driven HCC is associated with reduced motility and abnormal metabolic functions of CD8^+^ T cells rather than infiltration of CD8^+^ T cells and concentration of effector CD8^+^ T cells in tumor tissue (147, 148). Further researches need to be done to explore the reason why NASH-induced HCC is less sensitive to immunotherapy, which can not only broaden our horizons in the mechanism by which NASH promotes HCC but also help us to find targeted population of immunotherapy in HCC and investigate the feasibility of combined therapy in NASH-induced HCC.

## Roles of unconventional T cells

### NKT cells

Numerous NKT cells, a kind of innate-like cells, locate in liver sinusoids, expressing surface markers expressed by NK cells as well as TCR. NKT cells recognize lipid antigens dependent of CD1d (149). NKT cells are crucial to maintain the balance of immune system between inflammation and tolerance (150).

**Figure 3:**
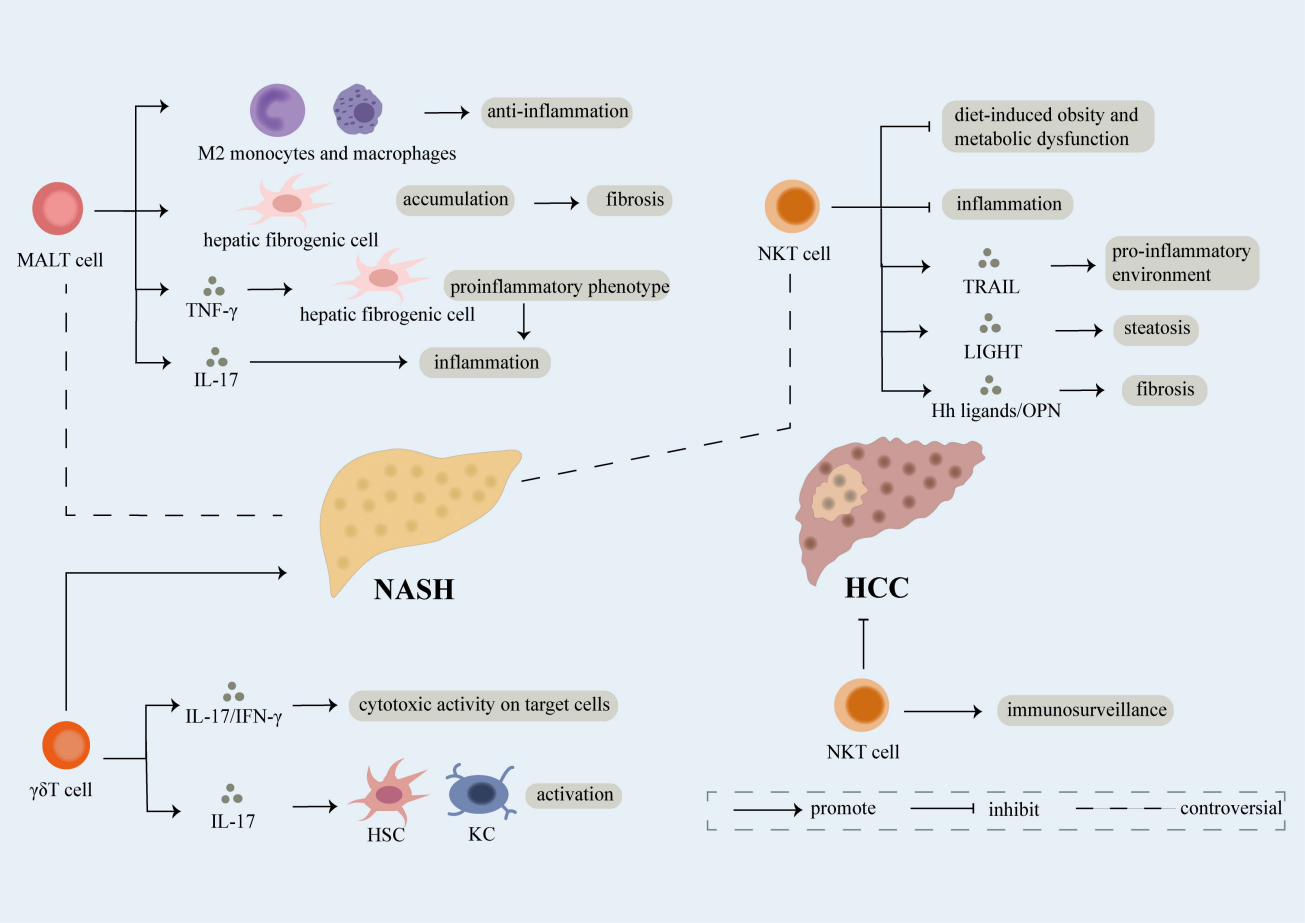
Role of unconventional T cell. Unconventional T cells like NKT, MALT, γδT cells participate in the development of NAFLD. γδT cells have been proven to facilitate the development of NAFLD through secretion of IL-17 and IFN-γ which can lead to cytotoxic activity on target cells. What’s more, IL-17 released by γδT cells can induce activation of hepatic stellate cells (HSCs) and KCs thus promoting fibrosis in NASH. However, conflicting results regarding the role of NKT and MALT cells in NAFLD have been exhibit. NKT cells have been reported as protective factors of NASH, mechanically, they can not only restrain diet-induced obesity and metabolic dysfunction but also limit inflammation. However, NKT cells can induce pro-inflammatory environment and steatosis dependent of TRAIL and LIGHT respectively. Meanwhile, fibrosis is accelerated partly attributed to release of Hh ligands/OPN by NKT cells. On one hand, MALT cells induce the differentiation of monocytes and macrophages into anti-inflammatory phenotype thus inhibiting development of NASH. On the other hand, MALT cells can not only induce the accumulation of hepatic fibrogenic cells which further result in fibrosis, but also promote inflammation through differentiation of hepatic fibrogenic cells into pro-inflammatory phenotype dependent of TNF-γ and release of IL-17. Last but not the least, NKT cells functioning as immune surveillance cells are crucial in suppressing NASH-induced HCC.

Researches have revealed that NKT cells restrain the aggravation of NAFLD (151). Depletion of NKT cells is uncovered in patient with NAFLD and diet-induced NAFLD mice (150, 151). High-fat diet induces increased pro-inflammatory KCs which further promote over-activation and cell death of NKT cells, thus leading to the deficiency of NKT cells in the progression of NAFLD (152). Tim-3^+^/Gal-9 pathway contributes to the depletion and secondary proliferation of NKT cells in diet-induced NAFLD (153). Mice lacking NKT cells exhibit higher rate of weight gain and liver steatosis which suggests that NKT cells involve in restricting obesity and metabolic dysfunction triggered by diet (154). CXCR6, a promotor of NKT cell recruitment, can restrict inflammation in NAFLD (155). Hepatic cholesterol accumulation selectively inhibits antitumor immunosurveillance of NKT cells in a diet-associated NAFLD-HCC mice model through lipid peroxide accumulation and deficient cytotoxicity in a SREBP2-dependent manner (156) (**Figure 3**).

However, conflicting concept exhibiting NKT cells facilitate NAFLD has been supported by several evidence. Infiltration of NKT cells has been proven in HFHC-induced progressive NASH (157), furthermore, NKT cells augment in liver and blood of patients with moderate to severe steatosis (158). NAFLD relies on iNKT cells in CDAA-fed mice, a growing number of CXCR3^+^IFN-γ^+^T-bet^+^IL-17A^+^ iNKT cells is uncovered in NASH patients (159). During convalescence of NASH, cytotoxic NKT cells decrease (160). NKT cells can promote proinflammatory environment and steatosis *via* secretion of (TNF)-related apoptosis-inducing ligand (TRAIL) and LIGHT, respectively (125, 161). What’s more, NKT cells are associated with NASH-related fibrosis. NKT cells are found to accumulate in cirrhotic livers. Activation of Hedgehog (Hh) pathway can induce recruitment of NKT cells in liver (162). Deficiency of NF-κB1 favors the recruitment of NKT cells through upregulation of IL-15, a cytokine regulating NKT cell survival and maturation (163). Hepatic NKT cells can activate HSCs to promote fibrosis through production of osteopontin (OPN) and Hh ligands (29, 164). Plasma concentrations of OPN are positively related to severity of liver fibrosis which may be a potential marker of NASH fibrosis (164) (**Figure 3**).

### MALT cells

MALT cells, are identified as CD3^+^CD4^-^CD161^hi^Vα7.2^+^ lymphocytes with a restricted species of β chain (mainly Vβ 6 and Vβ 20) (165, 166). The immunological surveillance function of MALT cells can be triggered by metabolites of vitamin B, an antigen presented by MR1 (167). The effector memory phenotype of MALT cells, responds to antigen relying on TCR and cytokine independent of TCR (166). The effect of MALT cells may attribute to production of proinflammatory cytokines containing IL-17, IFN-γ (168, 169) and releasement of cytotoxic factors like granzyme B and perforin (166).

MALT cells form 40% of intrahepatic T cells and involve in NAFLD-related diseases (166, 170). In patients with NAFLD, circulating MALT cells decrease and intrahepatic MALT cells increase. Functions of circulating MALT cells including rising secretion of IL-4 and reduced production of IFN-γ change. What’s more, activated MALT cells can induce differentiation of monocytes/macrophages into anti-inflammatory M2 phenotype. Taken together, MALT cells alleviate inflammation in NAFLD (171). However, it has been highlighted that MALT cells play a pro-inflammatory and pro-fibrotic role in NAFLD. MALT cells accumulate in liver fibrotic septa in patients suffering from NAFLD-related cirrhosis and exhibit a pro-inflammatory phenotype with increased IL-17^+^ T cells. Meanwhile, MALT cells can contract with hepatic fibrogenic cells and enhance the accumulation of hepatic fibrogenic cells in an MR-dependent manner to facilitate fibrosis. Meanwhile, MALT cells involve in promoting the transformation of hepatic fibrogenic cells to pro-inflammatory phenotype *via* TNF-γ thus accelerating inflammation (172) (**Figure 3**).

### γδT cells

γδT cells are characterized by a TCR γ chain and δ chain, and MHC-mediated antigen presentation is not required for their recognition of antigen (173). After antigen recognition, γδT cells can produce IL-17 and IFN-γ, exert cytotoxic effect on target cells, and activate other immune cells (174). Moreover, some studies suggest that γδT cells secrete even more IL-17 than classic Th17 cells in both adipose tissue and the liver (175, 176).

γδT cells are thought to promote the progression of NAFLD. In NASH mouse models, a marked elevation of γδT cells in both adipose tissue and liver has been verified, which is associated with development of NAFLD (176). The microbiota controls the number of γδT cells in the liver dependent of lipid antigen CD1d (177) and CCR2 can increase the infiltration of γδ T cells (177). Mice lacking γδT cells exhibit a significant attenuation in steatohepatitis after HFD treatment. And transfer of IL-17A^-/-^ rather than WT hepatic γδT cells display reduced NASH in mice with γδT cells deficiency, indicating that γδ T cells may contribute to NASH progression through IL-17 secretion (176). However, the pathogenic effect of γδT cells probably is independent of IL-17 (177). γδT cells can also facilitate fibrosis progression by activating HSC and Kupffer cells through production of IL-17 (73) (**Figure 3**).

## Therapeutic approaches

There are currently no clinically approved therapies for NASH (178). But considering the influence of T cells in the pathogenesis of NASH, therapies modulating them can conceivably prevent the progression of disease. The possible targets include recruitment of T cells into the liver, TCR signaling, proinflammatory cytokines secreted by T cells, survival and proliferation of specific T cell subsets. Various potential therapies have been tested in clinical trials.

Monocytes and T cells express CCR2 and CCR5, so cenicriviroc (CVC) which is a dual CCR2 and CCR5 antagonist can limit their infiltration in the liver and has been shown to limit fibrosis in NASH mice (179–181). However, results of the phase III study showed a lack of efficacy (12). Thus, to more efficiently limit NASH, CVC might be used with the combination of other agents blocking T cell recruitment (182). In NASH-driven HCC, although CCL2/CCR2 has a dual role on immune cells which can drive the infiltration of both MDSCs and CD4^+^ Th1, CD8^+^ cells (183), a pre-clinical study has demonstrated that a CCR2 antagonist which can block immunosuppression mediated by tumor-infiltrating macrophage and increase CD8^+^ T cells exhibits an anti-tumor role in HCC, with a more significant effect combined with low-dose sorafenib (184, 185).

Vedolizumab, the antibody against α4β7, which has already been approved for treating IBD, can block the binding of CD4^+^ T cells to MAdCAM-1 and result in inhibition of the pathogenic recruitment of CD4^+^ T cells and attenuate hepatic inflammation and fibrosis in a NASH model (186). The anti-MAdCAM-1 antibody is also in clinical trials (104). Either of them is a potential therapy for NASH.

T cells recognize antigens through TCR, which is associated with the CD3 molecule (187). A clinical trial of the mouse anti‐CD3 mAb OKT3 in NASH patients demonstrates that OKT3 can promote Treg cells with an increase in their numbers and secretion of anti‐inflammatory cytokines, indicating that OKT3 has the potential to inhibit the inflammatory process (188). What’s more, oral administration of OKT3 does not show immunosuppressive effects as it is not absorbed systemically, which is safer to use.

The stimulation of adenosine A2a receptor (A2aR) has been reported to reduce recruitment of Th1 and Th17 cells through the inhibition of CCL20 and CXCL10 expression and increase suppressive ability of Treg cells. Immunolipotoxicity induced by IL-17 is also prevented by A2aR activation *via* modulation of PTEN/PI3-kinase-Akt signaling (189). TGF-β which is a main profibrotic factor also decreases after the treatment. Taken together, A2aR stimulation is a hopeful therapeutic approach.

CXCL10 is identified as a key gene in NAFLD progression using a minimum depth random forest algorithm (190). The anti-CXCL10 antibody can reduce lipid accumulation and infiltration of inflammatory cells, consequently preventing steatohepatitis and reducing hepatic fibrosis. However, CXCR3^–/–^ mice exhibit more severe liver injury as CXCL9 exerts an anti-fibrotic effect. So treatment need to be highly specific for the CXCL10 (191). TNF-α secreted by T cells plays a pro-inflammatory role. The anti-TNF-α drug thalidomide and the neutralization of TNF-α by Infliximab both show decreased inflammation and fibrosis in NASH models (192, 193). TGF-β secreted by T cells mediates the activation of HSCs and is a key pro-fibrogenic cytokine (194). The anti-fibrotic TGF-β inhibitor Galunisertib can inhibit SMAD2 phosphorylation and block collagens deposition (195). Thus, the development of reagents to regulate the CXCL10/CXCR3 pathway, TNF-α and TGF-β may be useful therapeutic strategies to attenuate liver fibrosis.

Linoleic acid can upregulate carnitine palmitoyltransferase (CPT) gene which induces apoptosis of CD4^+^ T cells and promotes progression of HCC. Peroxisome proliferator-activated receptor alpha (PPAR-α) has been found to upregulate expression of CPT genes (196), but the exact role of PPAR-α in HCC is conflicting (197). Thus, CPT is a more ideal target and *in vivo* targeting of CPT blocks HCC development in NAFLD. Theaphenon E (TE), the green tea extract, significantly decreases the compensatory proliferation because of liver injury which promotes tumorgenesis, and increases survival of CD4^+^ T cell in HFD mice. The effect of TE may attribute to its inhibition of accumulation of linoleic acid in the liver (198). Oral administration of curcumin which is a polyphenol exerting antioxidant and anti-inflammatory effects, is proved to prevent HFD-induced liver injury, intrahepatic accumulation of CD4^+^ T cells and the pro-inflammatory effects on macrophages induced by linoleic acid (199).

Several studies also address the possibility of interfering with T cell-mediated responses as a novel approach for treating NASH, such as PR-957 which can hinder endothelial MHC-II antigen presentation to CD4^+^ T cells (200), lycopene,which can suppress the recruitment of T cells as well as activation of M1 macrophages (201), and koumine which can reduce the percentages of Th1 and Th17 cells and increase Th2 and Treg cells in the liver (202).

In conclusion, approaches targeting T cells is promising for future therapies.

## Conclusions and perspectives

NAFLD is a complicated disease caused by multifactor. Basic and clinical researches have both suggested that innate and adaptive immune activation plays a pivotal part in triggering and exacerbating hepatic inflammation in NAFLD, while persistent inflammation results in liver injury. We have described here how different T cell subsets exert diverse effects on liver inflammation and fibrosis, as well as the progression of NASH to HCC. However, current findings are conflicting regarding the exact roles T cells play in NAFLD. Our understanding is fragmented and requires further investigation. Mouse models of NASH need to be improved to closely mimic the human condition. Comparing the immune landscape in NASH patients with different mouse models could help to suggest a more superior preclinical model. Moreover, crosstalk between different immune cells and also between immune cells and HSCs also need to be considered. Although current knowledge indicates that there are opportunities to design new potential therapies or to improve current treatments regulating T cells and their responses, the clinical efficacy still remains to be demonstrated in more clinical settings. Considering that IL-17 can be produced by a variety of T cell subsets such as Th17, MALT and γδT cells and its critical role in NAFLD, the therapeutic potential of targeting IL-17 can be evaluated as a research goal in future. Indeed, antagonists of IL-17 have already been approved for the treatment of several autoimmune diseases such as IBD and RA (43). In addition, drugs such as statin, vitamin D, probiotics and retinoic acid, which can restore the balance of Th17/Treg cells, have already shown the potential to alleviate NASH (203). The imbalance of Th17/Treg cells, as an emerging biomarker for disease assessment and outcome prediction, may become a promising target and requires further exploration.

## Author contributions

TM and RY collected data and wrote this manuscript. YL and KH designed, reviewed, and revised this manuscript. All authors contributed to the article and approved the submitted version.

## Funding

This study was supported by the Project of the Shanghai Municipal Health Commission (20204Y0012), Seed Fund of Renji Hospital (RJZZ18-010), Shenkang three-year action plan (SHDC2020CR2003A, SHDC2020CR5012), Innovative Research Team of High-Level Local Universities in Shanghai (SSMU-ZDCX20180802), National Natural Science Foundation of China (81972205), and the Project of Shanghai key clinical specialties (shslczdzk05801).

## Conflict of interest

The authors declare that the research was conducted in the absence of any commercial or financial relationships that could be construed as a potential conflict of interest.

## Publisher’s note

All claims expressed in this article are solely those of the authors and do not necessarily represent those of their affiliated organizations, or those of the publisher, the editors and the reviewers. Any product that may be evaluated in this article, or claim that may be made by its manufacturer, is not guaranteed or endorsed by the publisher.

## Glossary

**Table:**

NAFLD	nonalcoholic fatty liver disease
NASH	nonalcoholic steatohepatitis
HCC	hepatocellular carcinoma
CD	cluster of differentiation
NAFL	nonalcoholic fatty liver
IR	insulin resistance
T2DM	type 2 diabetes mellitus
FFA	free fatty acid
SCGF-β	stem cell growth factor-beta
AT	adipose tissue
VAT	visceral adipose tissue
KC	kupffer cells
HSC	hepatic stellate cell
ROS	reactive oxygen species
NADPH	nicotinamide adenine dinucleotide phosphate
Th	helper T
STAT	signal transducing activator of transcription
TCPTP	T cell protein tyrosine phosphatase
TG	triglyceride
MCD	methionine- and cystine deficient diet
SFA	saturated fatty acid
GSK-3β	glycogen synthase kinase-3β
LPC	lysophosphatidylcholine
EV	extracellular vesicle
MUFA	monounsaturated fatty acid
ER	endoplasmic reticulum
TCR	T cell receptor
NKT	natural killer T
γδT	gamma delta T
MALT	mucosal-associated invariant T
Treg	regulatory T
IFN	Interferon
WT	wild-type
HOMA-IR	homeostasis model assessment-Insulin resistance
CRP	C-reactive protein
CCL	chemotactic cytokine
CXCL	chemokine (C-X-C motif) ligand
CXCR	chemokine (C-X-C motif) receptor
IL	i8nterleukin
IL-R	interleukin receptor
TNF	tumor necrosis factor
IMT	intima-media thickness
JNK	c-Jun NH-terminal kinase
PI3K	phosphoinositide-3 kinase
PTEN	phosphatase and tensin homologue
MT	metallothionein
VSMC	vascular smooth muscle cell
FOXP3	forkhead box protein 3
TGF	transforming growth factor
NET	neutrophil extracellular trap
DC	dendritic cell
LPS	lipopolysaccharide
HFD	high-fat diet
KLF10	kruppel-like factor 10
MIG	monokine Induced by Interferon-gamma
DEN	diethylnitrosamine
NAC	N-Acetylcysteine
URI	unconventional prefoldin RPB5 interactor
OS	overall survival
RAGE	receptor for advanced glycation product
Trm	tissue -resident memory T
CCR	chemokine receptor
FasL	factor related apoptosis ligand
Fas	factor related apoptosis
MHC	major histocompatibility complex
LTβR	lymphotoxin beta receptor
TAP	antigen peptide transporter
PD1	programmed cell death protein 1
PMN	polymorphonuclear neutrophil
MR	mineralocorticoid receptor
PD-L1	programmed cell death 1 ligand 1
ZNF24	zinc finger protein 24
Tc cells	cytotoxic T cells
CD-HFD	choline deficiency- high-fat diet
NF-κB	nuclear factor kappa-B
HBV	hepatitis B virus
HCV	hepatitis C virus
TAA	tumor-associated antigen
eTregs	effector regulatory T cells
CTLA	cytolytic T lymphocyte-associated antigen
IgA+	immunoglobulin-A-producing
TAM	tumor-associated macrophage
NRG	Neuregulin
Ncoa5	nuclear receptor coactivator 5
NK	natural killer
Tim-3	T cell immunoglobulin and mucin domain-containing protein 3
Gal-9	galectin-9
SREBP2	cholesterol regulatory element binding protein 2
iNKT	invariant natural killer T
CDAA	choline-deficient L-amino-defined diet
TRAIL	tumor necrosis factor-related apoptosis-inducing ligand
LIGHT	herpevirus entry mediator ligand
Hh	hedgehog
OPN	osteopontin
MR1	major histocompatibility complex class I-related
CVC	cenicriviroc
MDSC	myeloid-derived suppressor cells
MAdCAM-1	mucosal addressin cell adhesion molecule-1
mAb	monoclonal antibody
A2aR	adenosine A2a receptor
SMAD2	mothers against decapentaplegic homolog 2
CPT	carnitine palmitoyltransferase
TE	theaphenon E
IBD	inflammatory bowel disease
RA	rheumatoid arthritis
